# Outcomes of patients with multiple myeloma refractory to standard dose vs low dose lenalidomide

**DOI:** 10.1038/s41408-024-01039-1

**Published:** 2024-03-26

**Authors:** Utkarsh Goel, Charalampos Charalampous, Prashant Kapoor, Moritz Binder, Francis K. Buadi, David Dingli, Angela Dispenzieri, Amie Fonder, Morie A. Gertz, Wilson I. Gonsalves, Suzanne R. Hayman, Miriam A. Hobbs, Yi L. Hwa, Taxiarchis Kourelis, Martha Q. Lacy, Nelson Leung, Yi Lin, Rahma M. Warsame, Robert A. Kyle, S. Vincent Rajkumar, Shaji K. Kumar

**Affiliations:** 1 Division of Hematology, Mayo Clinic, Rochester, MN USA; 2 Department of Internal Medicine, Cleveland Clinic, Cleveland, OH USA

**Keywords:** Epidemiology, Myeloma

## Abstract

Refractoriness to lenalidomide is an important factor determining the choice of therapy at first relapse in multiple myeloma (MM). It remains debatable if resistance to lenalidomide varies among MM refractory to standard doses vs low dose maintenance doses. In this study, we assessed the outcomes with subsequent therapies in patients with MM refractory to standard dose vs low dose lenalidomide. We retrospectively reviewed all patients with MM at our institution who received first line therapy with lenalidomide containing regimens, and assessed progression free survival (PFS) and overall survival for these patients for second line therapy, and with lenalidomide retreatment. For second line therapy, we found no difference in the PFS between standard dose refractory and low dose refractory groups (median PFS 14 months vs 14 months, *p* = 0.95), while the PFS for both these groups was inferior to the not refractory group (median PFS 30 months, *p* < 0.001 for both pairs). Similar trends were seen among these groups on lenalidomide retreatment, and on multivariable analysis. These data suggest that refractoriness to lenalidomide is not dose dependent, and definition of lenalidomide refractoriness should not depend on the dose of lenalidomide to which the disease was considered refractory.

## Introduction

Lenalidomide containing regimens are extensively used as first line therapy for the treatment of multiple myeloma (MM) [1]. Since current treatment practice also relies on continuous therapy until progression or use of lenalidomide as maintenance therapy, most patients end up being refractory to lenalidomide at the time of first relapse. Hence, refractoriness to lenalidomide is an important consideration in determining the choice of therapy at first relapse [2].

It remains a point of debate whether lenalidomide resistance varies among patients who progress on standard/ full dose of lenalidomide (25 mg once daily) as compared to patients that progress on single agent maintenance doses (e.g., 5–15 mg once daily) [1, 3]. To this extent, often disease progressing on standard dose of lenalidomide is considered refractory to lenalidomide, while that progressing on maintenance doses is considered lenalidomide sensitive [4].

Defining refractoriness to lenalidomide is important, as disease that is considered refractory to lenalidomide would likely not benefit as much from lenalidomide based regimens and adopting a drug from a different class for second line therapy would be reasonable. On the other hand, lenalidomide sensitive disease may respond to an increased dose of lenalidomide, a new triplet regimen, or addition of another agent to the first line lenalidomide containing regimen [5, 6].

There are limited data assessing disease refractoriness to different doses of lenalidomide at first relapse, and the effect of retreatment with lenalidomide in these patients [3]. In this study, we sought to assess the impact of refractoriness to standard vs low doses of lenalidomide on outcomes with subsequent therapies in patients with MM.

## Methods

After approval from the Mayo Clinic Institutional Review Board, we retrospectively reviewed MM patients diagnosed between January 1, 2004, and December 31, 2018, who received first line therapy with lenalidomide containing regimens. Informed consent was obtained from all patients for review of their medical records. Baseline characteristics collected at diagnosis included age, gender, International Staging System (ISS) stage, and interphase Fluorescence in situ hybridization (FISH) abnormalities [7]. The mSMART criteria were used for risk stratification based on FISH abnormalities [8–10].

At the first relapse after diagnosis, we classified patients into 3 groups based on refractoriness to lenalidomide. 1. Patients with disease progressing on or within 60 days of receiving 25 mg once daily lenalidomide (Standard Dose Refractory group), 2. Patient with disease progression on or within 60 days of receiving 5 to 15 mg once daily lenalidomide (Low Dose Refractory group), and 3. Patients who received lenalidomide as a part of first line therapy and had disease progression after 60 days of stopping lenalidomide (Not Refractory group) [11]. At first relapse, we additionally assessed the disease as being refractory to proteasome inhibitors (PIs) or daratumumab. We classified second line regimens into the following mutually exclusive classes: daratumumab-based combinations, PI based combinations, combinations of PI and immunomodulatory drugs (IMiDs), and VDTPACE (bortezomib, dexamethasone, thalidomide, cisplatin, doxorubicin, cyclophosphamide, and etoposide)- like regimens. We also assessed whether patients were re-treated with a lenalidomide containing regimen after first line therapy at any point during the disease course. For this next lenalidomide containing line of therapy, we classified regimens into the following mutually exclusive groups: lenalidomide alone or in combination with dexamethasone, lenalidomide with daratumumab, lenalidomide with a PI, and lenalidomide with elotuzumab.

Baseline characteristics were summarized using descriptive statistics. We used the chi square test for comparing categorical variables and Kruskal-Wallis test for comparing continuous variables. We defined progression free survival (PFS) from time of start of therapy to disease progression or death, and overall survival (OS) as time from start of therapy to death due to all causes. PFS and OS were calculated using the Kaplan-Meier method, and differences between the groups were assessed using the log-rank test. Cox models were used to assess the prognostic significance of various parameters in predicting PFS and OS. Median follow up was calculated using the reverse Kaplan-Meier estimator method. For all tests, 2-sided *p*-values of < 0.05 were considered statistically significant. All analyses were performed using R version 4.2.1. De-identified data and R code used are available upon reasonable request to the corresponding author.

## Results

### Patient characteristics

A total of 476 patients were included in the study. Of these, 218 (45.8%) belonged to the Not Refractory group, 184 (38.7%) were in the Low Dose Refractory group, and 74 (15.5%) patients were in the Standard Dose Refractory group. Patients in the Low Dose Refractory group were slightly older (median age 65 years), as compared to Standard Dose Refractory (median age 63 years) and Not Refractory (median age 62 years) groups (*p* = 0.022). Other disease characteristics at diagnosis- including proportion of patients with ISS stage III disease, and high-risk FISH were comparable among the 3 groups (Table 1). Of the 218 patients in the Not Refractory group, 81 (37%) were not refractory to lenalidomide because they did not receive any maintenance therapy, 79 (36%) stopped maintenance therapy at least 60 days before first progression, and 58 (27%) patients received maintenance therapy with a proteasome inhibitor or daratumumab. Among the 184 patients in the Low Dose Refractory group, 164 (89%) were low dose refractory because they experienced disease progression on maintenance doses of lenalidomide, while the remaining 20 (11%) belonged to this group because they were administered low doses of lenalidomide due to adverse events to the standard lenalidomide dose.

**Table 1:** Baseline patient characteristics at diagnosis.

Parameters	All patients (*N* = 476) *N* (%)			
	Not Refractory Group (*n* = 218)	Low Dose Refractory Group (*n* = 184)	Standard Dose Refractory Group (*n* = 74)	*P*-value
Demographics				
Age, median (IQR)	61.8 (56.3–68.4)	65.4 (59.1–69.6)	63.3 (55.2–70.2)	0.022
Gender: Female	95 (43.5%)	80 (43.4%)	27 (36.4%)	0.52
eGFR < 60 mL/min/1.73 m^2^	43 (19.7%)	35 (19%)	18 (24.3%)	0.53
Disease characteristics				
ISS stage III	43 (19.7%)	35 (19%)	17 (22.9%)	0.64
FISH at diagnosis				
High risk	66 (30.2%)	55 (29.8%)	26 (35.1%)	0.80
t(4;14)	22 (10%)	6 (3.2%)	7 (9.4%)	
t(14;16)	4 (1.8%)	6 (3.2%)	4 (5.4%)	
t(14;20)	1 (0.4%)	4 (2.1%)	1 (1.3%)	
del(17p)	24 (11%)	13 (7%)	8 (10.8%)	
gain(1q)	34 (15.5%)	37 (20.1%)	10 (13.5%)	
M-protein isotype				
IgG	124 (56.8%)	101 (54.8%)	42 (56.7%)	0.91
IgA	45 (20.6%)	44 (23.9%)	14 (18.9%)	0.60
Light chain only disease	38 (17.4%)	28 (15.2%)	15 (20.2%)	0.60
Other/ Not available	11 (5.04%)	11 (5.9%)	3 (4.05%)	
First line therapy received				
Doublet	85 (38.9%)	61 (33.1%)	40 (54%)	0.007
Triplet	120 (55.0%)	121 (65.7%)	34 (46%)	0.007
Quadruplet	13 (5.9%)	2 (0.1%)	0 (0%)	0.004
First line ASCT	160 (73.3%)	92 (50%)	18 (24.3%)	< 0.001

*IQR* Indicates interquartile range, *ISS* International Staging System, *FISH* Fluorescence in situ hybridization, *t* translocation, *del* deletion, *ASCT* Autologous stem cell transplantation.

### Second line therapy and next lenalidomide containing therapy

Treatments received as second line therapy and in the next lenalidomide-containing line of therapy are described in Table 2. A higher proportion of patients in the Low Dose Refractory group (43.4%) were treated with daratumumab based regimens (*p* = 0.003). A higher proportion of patients in the Standard Dose Refractory group received an autologous stem cell transplant (ASCT) (39.1%) or VDTPACE-like regimens (6.7%) as a part of the second line therapy. Patients who were not refractory to lenalidomide at first relapse were treated with IMiD based combinations more commonly as compared to the lenalidomide refractory groups (*p* = 0.001). Of the total 476 patients, 212 (44.5%) patients were re-treated with a lenalidomide containing regimen at any point during the disease course. Of these 212 patients, 140 (66%) were not refractory to lenalidomide at their first relapse, 43 (20%) were refractory to low doses of lenalidomide, and 29 (14%) were refractory to standard dose of lenalidomide at first relapse.

**Table 2:** Treatments received at first relapse and in next lenalidomide containing regimen.

Treatments received at first relapse				
	Not Refractory Group (*n* = 218)	Low Dose Refractory Group (*n* = 184)	Standard Dose Refractory Group (*n* = 74)	*P*-value
Daratumumab based combinations	76 (34.8%)	80 (43.4%)	16 (21.6%)	0.003
PI based combinations	58 (26.6%)	65 (35.3%)	27 (36.4%)	0.10
PI and IMiD based combinations	38 (17.4%)	24 (13.04%)	14 (18.9%)	0.36
IMiD based combinations	32 (14.6%)	8 (4.3%)	6 (8.1%)	0.001
VDTPACE like regimens	3 (1.3%)	2 (1.08%)	5 (6.7%)	0.009
Other regimens	11(5.04%)	5 (2.7%)	6 (8.1%)	0.16
ASCT at first relapse	18 (8.2%)	28 (15.2%)	29 (39.1%)	< 0.001
**Treatments received in next lenalidomide containing regimen**				
	**Not Refractory Group (*n*** = **140)**	**Low Dose Refractory Group (*n*** = **43)**	**Standard Dose Refractory Group (*n*** = **29)**	***P*-value**
R alone/ with dexamethasone	26 (18.5%)	4 (9.3%)	1 (3.4%)	0.06
R with daratumumab	53 (37.8%)	10 (23.2%)	11 (37.9%)	0.19
R with PI	40 (28.5%)	23 (53.4%)	14 (48.2%)	0.004
R with elotuzumab	12 (8.5%)	1 (2.3%)	2 (6.8%)	0.37
Other combinations	9 (6.4%)	5 (11.6%)	1 (3.4%)	0.36
ASCT	11 (7.8%)	3 (6.9%)	4 (13.7%)	0.14

R indicates lenalidomide, *PI* Proteasome inhibitor, *IMiD* Immunomodulatory drug, *VDTPACE* Bortezomib, dexamethasone, thalidomide, cisplatin, doxorubicin, cyclophosphamide, and etoposide, *ASCT* Autologous stem cell transplantation.

### Outcomes for second line of therapy

**Fig. 1:**
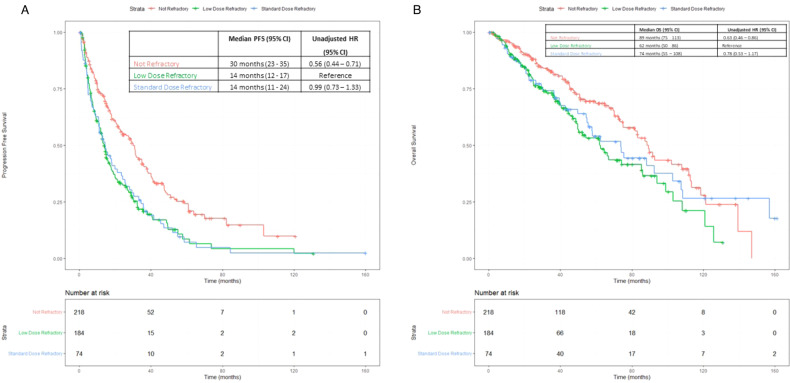
Outcomes for second line of therapy. **A** Progression free survival in 2nd line (immediate next line) of therapy. **B** Overall survival from 2nd line (immediate next line) of therapy.

For the entire cohort, the median follow-up from diagnosis was 110 months (95% CI: 100–120 months), and from first relapse was 56 months (95% CI: 51– 62 months). The median time from diagnosis to first relapse for the entire cohort was 33 months (interquartile range (IQR) 20–53 months). The median time from diagnosis to first relapse for the Not Refractor group was 36 months (IQR 25–55 months), for the Low Dose Refractory group was 32 months (IQR 19–50 months), and for the Standard Dose Refractory group was 20 months (IQR 7–45 months). For second line of therapy, the median PFS for the Standard Dose Refractory group (14 months, 95% CI: 11–24 months) did not significantly differ when compared to the Low Dose Refractory group (median PFS 14 months, 95% CI: 12–17 months, *p* = 0.95). The PFS for both Standard Dose and Low Dose Refractory groups was inferior as compared to the Not Refractory group (median PFS 30 months, *p* < 0.001 for both pairs) (Fig. 1A). These differences were maintained after adjusting for high-risk FISH at diagnosis, ISS stage, additional refractoriness to a PI or daratumumab, type of regimen received, and receipt of ASCT at relapse. On multivariable analysis, the hazard ratio (HR) for PFS for the Low Dose Refractory group was 2.13 (95% CI: 1.56–2.9), while that for the Standard Dose Refractory group was 2.08 (95% CI: 1.42–3.0), as compared to the Not Refractory group (HR = 1) (Supplementary Fig. 1).

From the first relapse, the median OS for the Not Refractory group was 89 months (95% CI: 75–113 months), as compared to 74 months (95% CI: 55–108 months) for the Standard Dose Refractory group (*p* = 0.38) and 62 months (95% CI: 50–86 months) for the Low Dose Refractory group (*p* = 0.006). There was no OS difference between the Standard Dose Refractory and Low Dose Refractory groups (*p* = 0.40) (Fig. 1B). After adjusting for parameters as listed above, there were no significant OS differences noted among the groups. The HR for OS was 0.1.50 (95% CI: 0.99–2.27) for the Low Dose Refractory group, and 1.23 (95% CI: 0.74– 2.05) for the Standard Dose Refractory group, as compared to the Not Refractory group (Supplementary Fig. 2).

### Outcomes for the next lenalidomide containing line of therapy

Of the total 476 patients, a total of 212 patients (44.5%) were re-treated with a lenalidomide containing regimen at any point during the disease course, as described earlier. Of these 212, for 121 patients the next lenalidomide-containing line of therapy was also the second line (immediate next line) of therapy. Of the remaining 91 patients, the median time to next lenalidomide-containing regimen after the first relapse was 23 months.

**Fig. 2:**
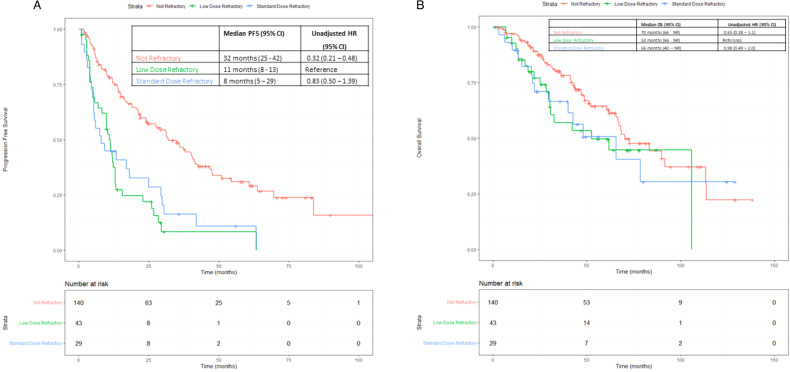
Outcomes with lenalidomide retreatment. **A** Progression free survival in next lenalidomide-containing line of therapy. **B** Overall survival from next lenalidomide containing line of therapy.

For the next lenalidomide-containing line of therapy after first relapse, the median PFS for the Standard Dose Refractory group was 8 months (95% CI: 5–29 months) and was not significantly different as compared to the Low Dose Refractory group (median PFS 11 months, 95% CI: 8–13 months, *p* = 0.62). The PFS for both Standard Dose and Low Dose Refractory groups was inferior to the Not Refractory group (median PFS 32 months, 95% CI: 25–42 months, *p* < 0.0001 for both pairs) (Fig. 2A). These differences were maintained after adjusting for ISS stage, presence of high-risk FISH, type of lenalidomide containing regimen received, and receipt of ASCT at relapse. The adjusted HR for PFS for the Low Dose Refractory group was 3.97 (95% CI: 2.23– 6.8), and HR for PFS for the Standard Dose Refractory group was 4.21 (95% CI: 2.30–7.7), as compared to the Not Refractory Group (HR = 1) (Supplementary Fig. 3).

From the next lenalidomide-containing line of therapy, the median OS for the Not Refractory group was 70 months, as compared to 66 months for the Standard Dose Refractory, and 53 months for the Low Dose Refractory group; however, the OS did not significantly differ among the groups (*p* = 0.97 for Standard Dose vs Low Dose Refractory, *p* = 0.24 for other two pairs) (Fig. 2B). Similarly, the OS did not differ among the 3 groups on multivariable analysis adjusting for parameters as above (Supplementary Fig. 4).

Since the presence of renal impairment might affect the effective dose of lenalidomide, we conducted an additional analysis excluding patients with known renal impairment at diagnosis, i.e. eGFR <60 mL/min/1.73 m^2^ (*n* = 96). In the remaining cohort of 380 patients, 149 (39%) were Low Dose Refractory, 56 (15%) were Standard Dose Refractory, and 175 (46%) were Not Refractory at the first relapse. 174 of these 380 patients were re-treated with lenalidomide at any point during their disease course. Of these 174 patients, 37 (21%) belonged to the Low Dose Refractory group, 19 (11%) belonged to the Standard Dose Refractory group, and 118 (68%) belonged to the Not Refractory group. For the second line therapy, following the trends in the overall cohort, the median PFS for the Standard Dose Refractory group (14 months, 95% CI: 9–26 months) did not significantly differ when compared to the Low Dose Refractory group (median PFS 14 months, 95%CI: 12–17 months, *p* = 0.67). The PFS for both Standard Dose and Low Dose Refractory groups was inferior to the Not Refractory group (median PFS 31 months, 95% CI: 24–38 months, *p* < 0.01 for both pairs). Similarly for the next lenalidomide containing line of therapy, the median PFS for the Standard Dose Refractory group (9 months, 95% CI: 5-NR) did not differ when compared to the Low Dose Refractory group (median PFS 11 months, 95% CI: 8–13 months, *p* = 0.54). The PFS for both Standard and Low Dose Refractory groups was inferior to the Not Refractory group (median PFS 36 months, 95% CI: 27–47 months, *p* < 0.001 for both pairs). In terms of overall survival for second line therapy, the OS for the Not Refractory group was 89 months (95% CI: 81-118 months), as compared to 58 months (95% CI: 50- NR) for the Standard Dose Refractory group (*p* = 0.13) and 62 months (95% CI: 50–103 months) for the Low Dose Refractory group (*p* = 0.02). There was no OS difference between the Standard Dose and Low Dose Refractory groups (*p* = 0.96).

From the first relapse, the median OS for the Not Refractory group was 89 months (95% CI: 75–113 months), as compared to 74 months (95% CI: 55–108 months) for the Standard Dose Refractory group (*p* = 0.38) and 62 months (95% CI: 50–86 months) for the Low Dose Refractory group (*p* = 0.006). There was no OS difference between the Standard Dose Refractory and Low Dose Refractory groups (*p* = 0.40). For the next lenalidomide containing line of therapy, the median OS for the Not Refractory group was 72 months, as compared to 52 months for the Low Dose Refractory and 43 months for the Standard Dose Refractory group; however the OS did not significantly differ among the groups (*p* = 0.71 for Standard vs Low Dose Refractory, *p* = 0.12 for Low Dose Refractory vs Not Refractory, *p* = 0.1 for Standard Dose Refractory vs Not Refractory).

## Discussion

In this retrospective study, we sought to assess the impact of refractoriness to standard dose vs low doses of lenalidomide on outcomes with subsequent therapies in patients with MM. We found that the PFS for 2nd line therapy, and with lenalidomide retreatment, did not differ between patients who were refractory to standard dose lenalidomide vs those refractory to low doses of lenalidomide. These trends were maintained on multivariable analysis.

These findings are important for determining the choice of regimen at first relapse in MM. Since the disease being refractory or sensitive to lenalidomide is a major consideration in deciding the choice of second line regimen, this study suggests that the definition refractoriness to lenalidomide should not be dependent on the dose of lenalidomide to which the disease was considered refractory. Patients progressing on lower maintenance doses (5–15 mg) should be considered as refractory to lenalidomide as patients progressing on the 25 mg dose.

A similar retrospective study by Kastritis et al. assessed 147 patients with relapsed/refractory multiple myeloma treated with pomalidomide and dexamethasone, who had been exposed or refractory to lenalidomide at any prior point in the disease course. Based on the last dose of lenalidomide, they found that the PFS and OS did not differ among patients with the last dose of lenalidomide being 25 mg vs 5–15 mg [3]. The present study using a cohort of patients receiving standard of care confirms these findings and adds to the literature. In the current study, 258 patients were refractory to lenalidomide in some form and 218 were not refractory to lenalidomide at first relapse. In addition, we were able to assess the effect of retreatment with lenalidomide in patients who had been exposed to lenalidomide as first line therapy, as well as patients who were refractory to lenalidomide at the first relapse.

We did not find differences in PFS when patients with disease refractory to standard vs low doses of lenalidomide were re-treated with lenalidomide containing regimens. The lack of difference between Standard Dose Refractory and Low Dose Refractory groups with lenalidomide re-treatment might be due to small numbers in the respective groups in the current study. Even so, the PFS for the Not Refractory group was significantly different from both the other groups, supporting the idea that although Low Dose Refractory disease might still respond to lenalidomide retreatment, the outcomes are significantly different to not consider these patients as sensitive to lenalidomide as patients in the Not Refractory Group. We did not find differences in overall survival among the 3 groups on multivariable analysis, which could be attributed to unaccounted for differences in subsequent therapies received after the second line therapy.

Our study is prone to inherent limitations due to its retrospective nature and data being from a single institution, which could introduce biases in terms of the patient population, availability of therapies, and practice patterns. Our study comprises of a heterogenous cohort in terms of treatments received at first relapse, and in the next lenalidomide containing line of therapy. This might limit comparison across the 3 groups, even after adjusting for the type of regimen received. We also did not assess the impact of increasing the dose of lenalidomide or reintroduction of dexamethasone within the same line of therapy once progression is noted while receiving lenalidomide. This might be the predominant pattern of practice and should be studied to better understand lenalidomide resistance in MM. Future studies should also assess the impact of resistance to different doses and schedules of other MM drugs like PIs and daratumumab.

In conclusion, the PFS for second line therapy and the next lenalidomide containing line of therapy did not differ between MM refractory to standard dose of lenalidomide vs low doses of lenalidomide at first relapse. The PFS for both these groups were significantly inferior to disease that was not refractory to lenalidomide at first relapse. These data suggest that clinical resistance to lenalidomide is not dose dependent, and definition of lenalidomide refractoriness should not depend on the dose of lenalidomide to which the disease was considered refractory.

### Supplementary information

Supplemental material

## Data Availability

De-identified data are available upon request to the corresponding author.
