# Supplementary material for: Outcomes of patients with multiple myeloma refractory to standard dose vs low dose lenalidomide
Source: Blood Cancer J. 2024 Mar 26;14(1):55. doi: 10.1038/s41408-024-01039-1 (PMC10966094; doi:10.1038/s41408-024-01039-1)
Supplement: Supplementary file 1 — Supplemental material [file 41408_2024_1039_MOESM1_ESM.pdf]

Supplementary Material

1. Supplementary Figure 1- Cox model for PFS for 2nd line (immediate next line) of therapy
2. Supplementary Figure 2- Cox model for OS for 2nd line (immediate next line) of therapy
3. Supplementary Figure 3- Cox model for PFS for next lenalidomide containing line of therapy
4. Supplementary Figure 4- Cox model for OS for next lenalidomide containing line of therapy

Supplementary Figure 1- Cox model for PFS for 2nd line (immediate next line) of therapy

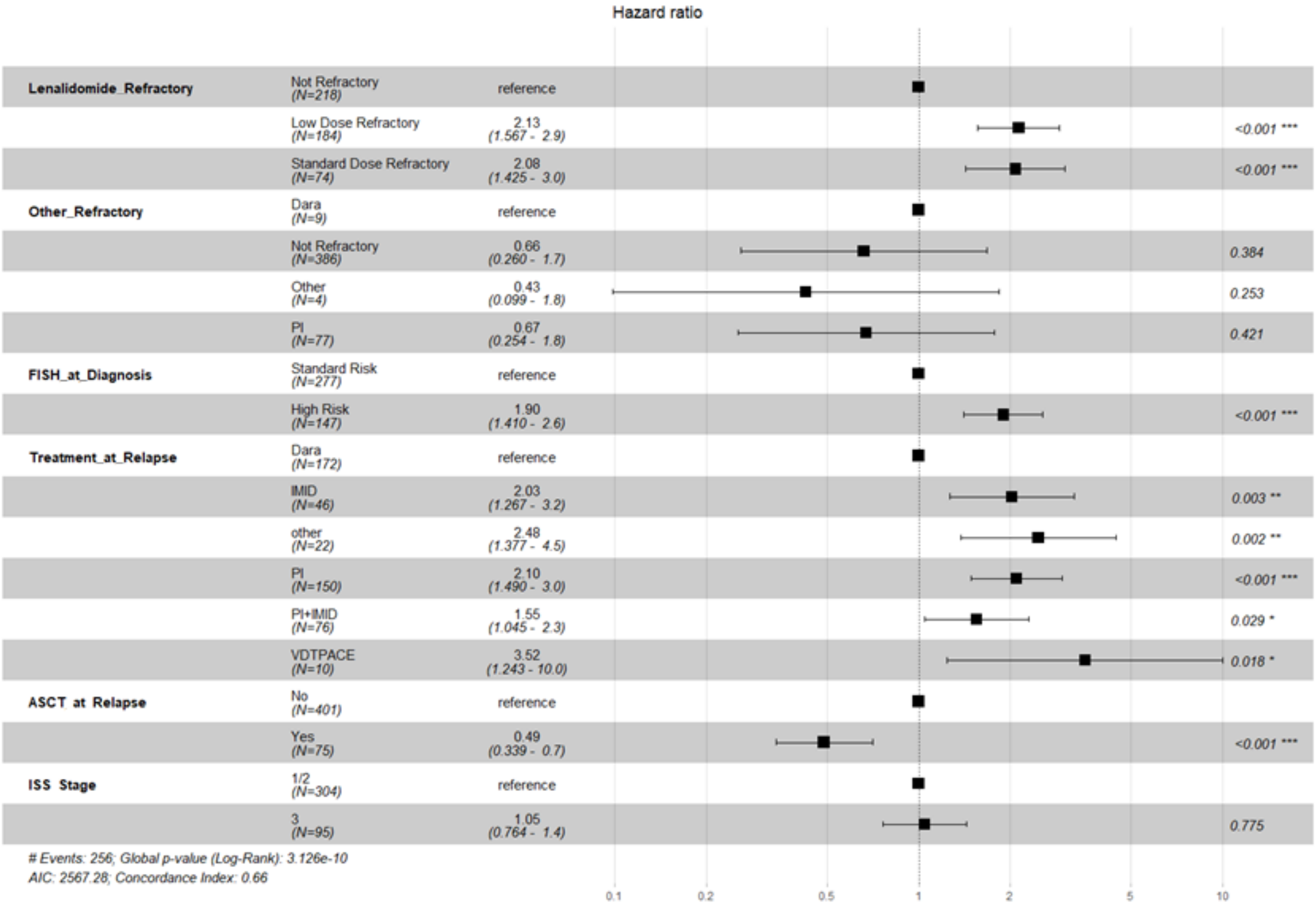

PFS indicates progression free survival; Dara, daratumumab; PI, proteasome inhibitor; FISH, fluorescence in-situ hybridization; IMID, immunomodulatory drug; VDTPACE, bortezomib, dexamethasone, thalidomide, cisplatin, doxorubicin, cyclophosphamide, etoposide; ASCT, autologous stem cell transplantation; ISS, international staging system.

Supplementary Figure 2- Cox model for OS for 2nd line (immediate next line) of therapy

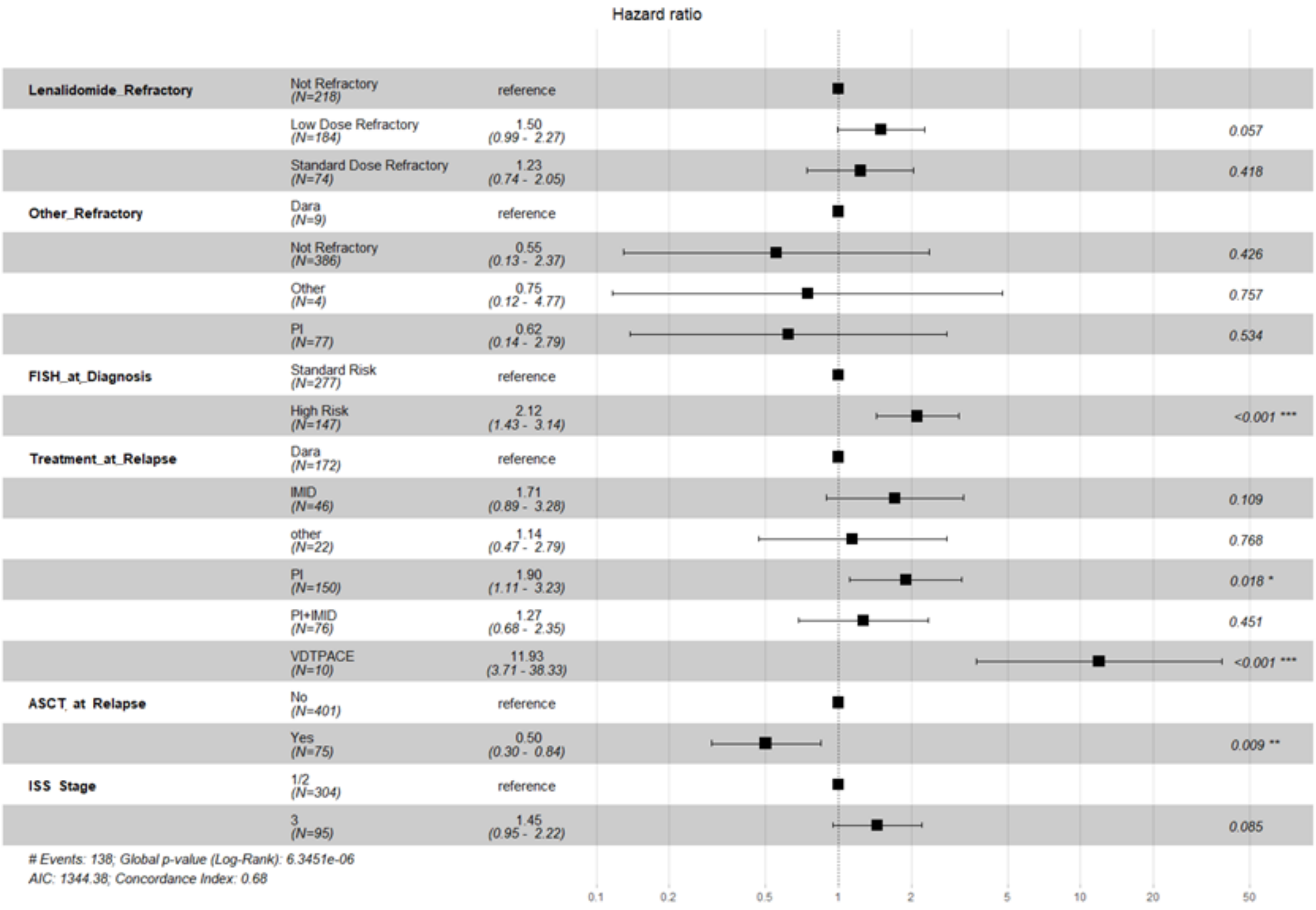

OS indicates overall survival; Dara, daratumumab; PI, proteasome inhibitor; FISH, fluorescence in-situ hybridization; IMID, immunomodulatory drug; VDTPACE, bortezomib, dexamethasone, thalidomide, cisplatin, doxorubicin, cyclophosphamide, etoposide; ASCT, autologous stem cell transplantation; ISS, international staging system.

Supplementary Figure 3- Cox model for PFS for next lenalidomide containing line of therapy

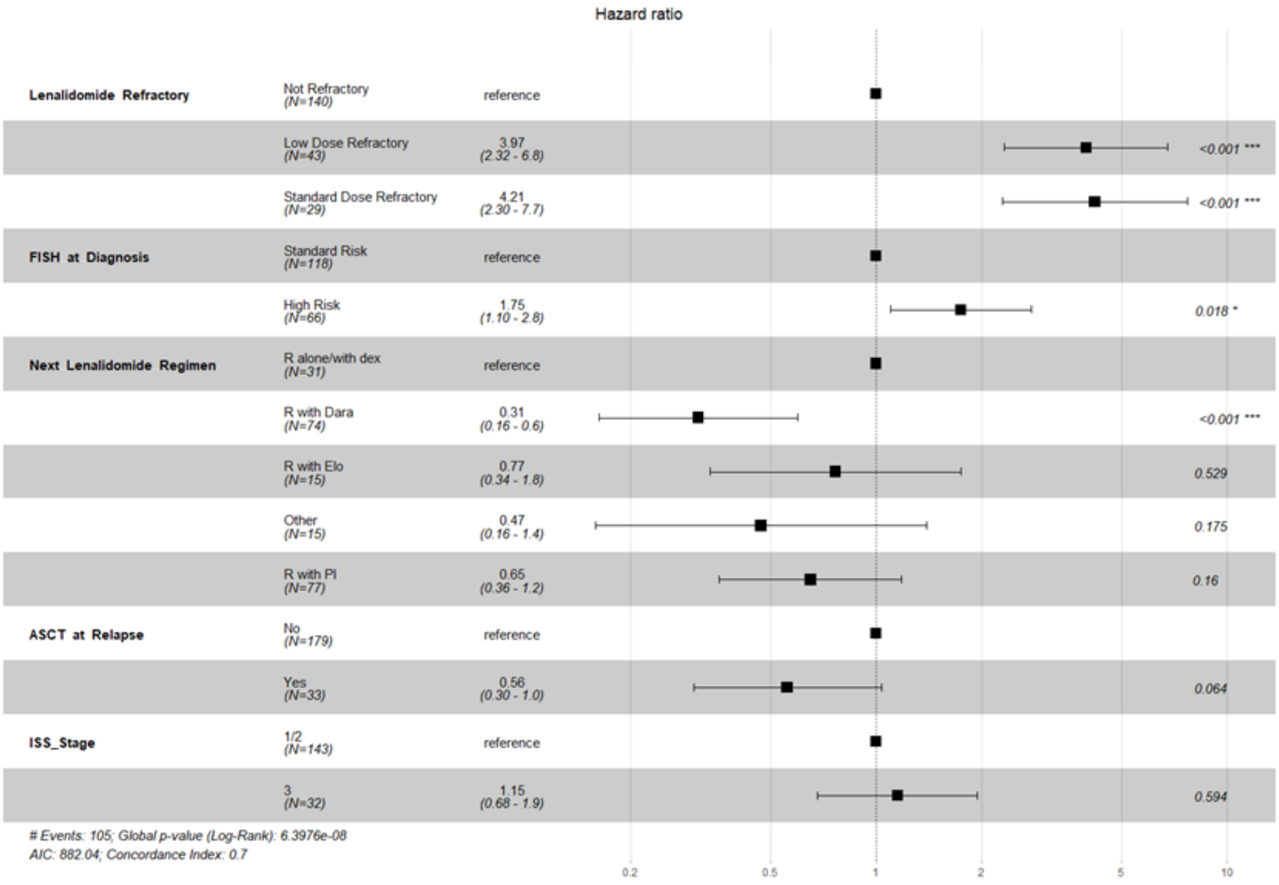

PFS indicates progression free survival; FISH, fluorescence in-situ hybridization; R, lenalidomide; dex, dexamethasone; Dara, daratumumab; Elo, elotuzumab; PI, proteasome inhibitor; ASCT, autologous stem cell transplantation; ISS, international staging system.

Supplementary Figure 4- Cox model for OS for next lenalidomide containing line of therapy

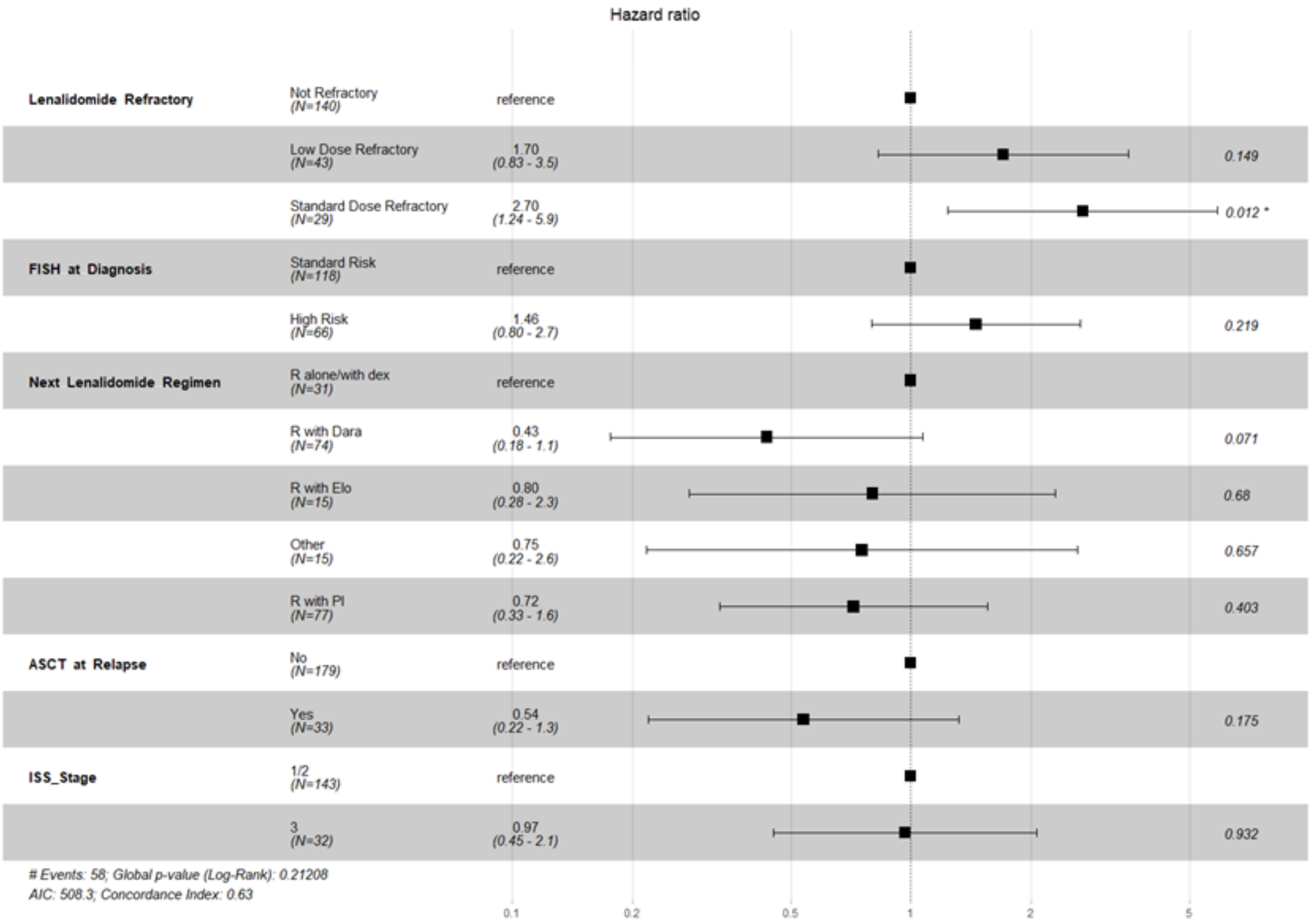

OS indicates overall survival; FISH, fluorescence in-situ hybridization; R, lenalidomide; dex, dexamethasone; Dara, daratumumab; Elo, elotuzumab; PI, proteasome inhibitor; ASCT, autologous stem cell transplantation; ISS, international staging system.
